# Open-Source Benchmarking of Plant-Based and Animal Meats

**DOI:** 10.3390/foods15122112

**Published:** 2026-06-11

**Authors:** Sybren D. van den Bedem, Ellen Kuhl, Caroline Cotto

**Affiliations:** 1 Department of Mathematics, Stanford University, Stanford, CA 94305, USA; syb@stanford.edu; 2 Department of Mechanical Engineering, Stanford University, Stanford, CA 94305, USA; 3 NECTAR, Food System Innovations, Claremont, CA 91711, USA; caroline.cotto@nectar.org

**Keywords:** sensory evaluation, consumer acceptance, plant-based meat, alternative protein, overall liking, texture perception

## Abstract

Global food production must reduce environmental impact while meeting rising demand for dietary protein. Plant-based meats aim to preserve the sensory and cultural role of animal meat while lowering greenhouse gas emissions, land use, and health risks. Advances in protein structure and flavor chemistry have improved product quality, yet consumers continue to prioritize taste and texture over sustainability, and systematic large-scale consumer surveys are scarce. It remains unclear how plant-based products rank against animal benchmarks and which product attributes most strongly influence overall liking. Here we show, in a large-scale blinded in-person sensory evaluation across 14 product categories, 2684 consumers, more than 11,000 product evaluations and 800,000 data points, that plant-based products still trail animal benchmarks at the category average level but approach parity in selected formats. Plant-based unbreaded chicken filets, chicken nuggets, and burgers achieved mean overall liking scores of 5.1, 4.9, and 5.2, differing from the animal benchmarks by only Δ = 0.1, 0.2, and 0.3 points on a seven-point scale. For unbreaded chicken filets and burgers, 48% and 47% of the participants rated the plant-based product the same as or better than the animal benchmark. Categories with higher sensory parity captured 5–14% market share compared with less than 1% for low-parity categories. Penalty analysis identified savoriness, aftertaste, juiciness, and tenderness as the strongest determinants of liking. These findings show that sensory parity is technically achievable but not yet consistent across product types. By publicly sharing all the sensory, preference, and market-linked data, we establish an open benchmark for alternative protein performance to democratize research and accelerate principled data-driven innovation.

## 1. Motivation

The global food system approaches a breaking point. Decades of research have already established that food production generates roughly one third of anthropogenic greenhouse gas emissions and accelerates deforestation, freshwater depletion, and biodiversity loss [1]. The global demand for food will rise sharply by mid-century, intensifying pressure on already stressed ecosystems [2]. Current dietary trajectories push planetary systems beyond safe operating limits and jeopardize climate stabilization goals [3]. These facts no longer surprise; they define the baseline of contemporary food science. What remains unresolved is how to translate this knowledge into scalable dietary change.

**Animal agriculture amplifies environmental and health risk.** Livestock production contributes substantially to greenhouse gas emissions, land degradation, and water scarcity [4]. Beef production alone carries a carbon footprint many times higher than most plant-based protein sources [3]. Diets high in red and processed meat associate with elevated risks of cardiovascular disease, type 2 diabetes, and colorectal cancer [5]. Modeling studies show that widespread dietary shifts toward plant-forward patterns could reduce food-related greenhouse gas emissions by up to 70% while improving global health outcomes [6]. Policymakers increasingly recognize dietary transition as essential for meeting Paris Agreement targets [7]. Yet, societies will not abandon meat unless alternatives satisfy deeply rooted expectations for taste, texture, and culinary experience [8].

**Plant-based meats redefine meat beyond the animal.** Plant-based products seek to preserve the sensory and cultural role of meat while reducing the environmental burden of animal agriculture [9]. Modern formulations combine structured plant proteins, fats, binders, and flavor systems to approximate muscle architecture and mouthfeel [10]. High-moisture extrusion and shear-cell technologies now generate fibrous textures that increasingly resemble animal muscle tissue [11]. Life-cycle assessments demonstrate substantial reductions in greenhouse gas emissions, land use, and water use relative to conventional beef [12]. However, sensory performance varies markedly across products and categories [13]. Although many consumers express concerns about environmental sustainability, flavor and texture remain the primary determinants of food choice and drive preference for animal meat over plant-based alternatives [14]. Researchers seek to address this sensory gap by linking measurable mechanical properties [15] to perceived texture [16], but product development still relies largely on iterative reformulation rather than systematic transparent benchmarking against animal counterparts [17].

**Consumers remain skeptical.** Adoption depends on sensory satisfaction, not environmental intent [18]. Many consumers perceive plant-based meats as inferior in savoriness, juiciness, and overall flavor depth [19]. Sensory analyses reveal that meat is consistently associated with positive terms, whereas meat alternatives are viewed more negatively [20]. These findings clearly define the performance gap [21], and major food manufacturers invest heavily in reformulation efforts to close it. Yet, most large-scale sensory datasets remain proprietary and siloed within companies, which limits independent validation, cross-category comparison, and cumulative scientific progress [22]. This fragmentation obscures true performance, blunts accountability, and slows the path toward sensory parity [23].

Here we address these limitations by conducting a large-scale blinded sensory comparison of plant-based and animal meats across multiple categories to establish a rigorous and transparent benchmark of current performance [24]. We share all the sensory data and analyses as an open-source resource to democratize food science and accelerate discovery and innovation toward sustainable protein systems [25].

## 2. Methods

We conducted a large-scale blinded in-person sensory study to compare plant-based meat products with conventional animal benchmarks across 14 product categories between November 2024 and January 2025 [25]. Palate Insights conducted the sensory study under informed consent procedures and applicable privacy safeguards as part of the NECTAR Taste of the Industry 2025 analysis; the present work analyzed the resulting fully de-identified open-source dataset made publicly available by NECTAR. To reflect real-world consumption contexts we performed all tests at restaurant partner locations in San Francisco, CA, and New York City, NY. We prepared all products according to manufacturer instructions and allowed participants to add condiments when appropriate. We implemented a blinded randomized within-subject design, where participants evaluated one product at a time. Each participant evaluated six products of the same category under blinded conditions, five plant-based and one animal-based one. We randomized product presentation order within each session to minimize order and sensory fatigue effects. After tasting each product, participants completed a standardized survey that captured sensory evaluations, similarity to conventional products, purchase intent, and open-ended feedback questions. Before tasting the next product, participants rinsed with water to reduce carryover effects. The full study included 2684 participants, more than 11,000 plant-based product evaluations, and more than 800,000 data points.

### 2.1. Tested Products

**Figure 1:**
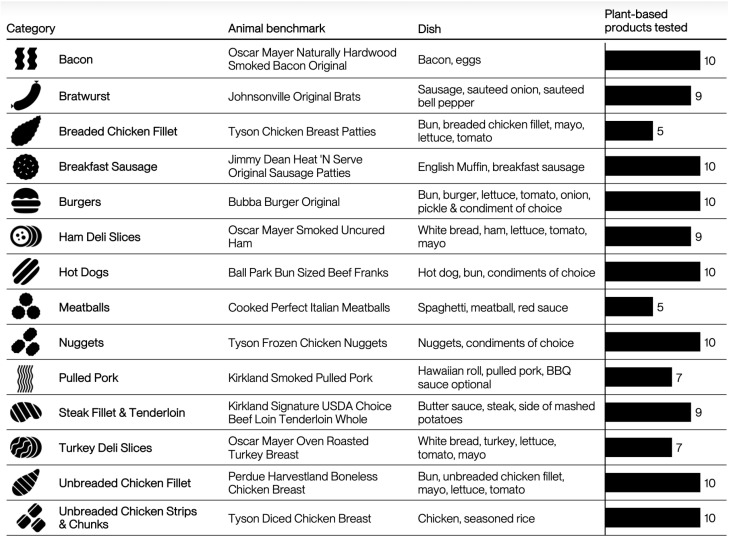
**Tested products.** This study tested 14 product categories with a total 121 plant-based products and 14 animal benchmarks. We selected the 14 product categories by high sales volume and market development and the 121 plant-based products by market presence, market readiness, animal-analog design, animal-compatible flavor, and distinct ingredient or production technology. We served each product in a complete but simplified product-specific dish.

We selected fourteen product categories based on two criteria: high sales volume and sufficient market development with at least five plant-based products available within the category (Figure 1). The study targeted commercially prominent and broadly representative categories rather than exhaustive coverage of all available plant-based products. Within these categories, we selected 121 plant-based products, 5–10 per category (mean 8.6) based on significant market presence, market-ready status, design as an animal analog, original flavor comparable to the animal product, and distinct ingredients or production technologies relative to other products tested within the category. We determined market presence using publicly available retail sales rankings, distribution across major US retailers and food-service channels, and industry category analyses from NECTAR. For each category, we selected a conventional animal benchmark based on highest retail sales volume. These criteria ensured that both plant-based and animal products reflected the commercially relevant market landscape at the time of testing. The fourteen categories include bacon, bratwurst, breaded chicken filets, breakfast sausages, burgers, chicken nuggets, deli ham, deli turkey, hot dogs, meatballs, pulled pork, steak, unbreaded chicken filets, and unbreaded chicken strips. We served each item as a complete but simplified build that matched typical consumption formats for the category.

### 2.2. Study Population

**Figure 2:**
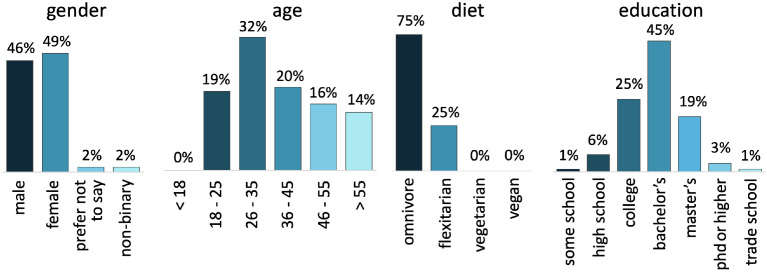
**Study population.** Demographic overview of the 2684 study participants. We enrolled a gender-, age-, and educationally balanced population of omnivores and flexitarians to ensure that all participants actively consumed animal meat.

The study population consists of 2684 US consumers, of whom 75% identified as omnivores and 25% as flexitarians (Figure 2). The gender distribution includes 49% female, 46% male, 2% non-binary, and 2% who preferred not to disclose. The age distribution includes 19% 18–25 years old, 32% 26–35, 20% 36–45, 16% 46–55, and 14% over 55. The study population had a diverse education background, with 6% holding a high school degree, 25% a college degree, 45% a bachelor’s degree, 19% a master’s degree, 3% a PhD or higher, and 3% some other education. We excluded vegetarians and vegans to ensure that all participants actively consumed animal meat and can provide meaningful benchmark comparisons.

### 2.3. Data Analysis

Participants rated overall liking, flavor, texture, and appearance on a seven-point Likert scale ranging from dislike very much (1) to like very much (7) and rated purchase intent from definitely would not buy (1) to definitely would buy (7). We calculated mean scores for each product and compared three benchmark groups, the *plant-based leader*, defined as the highest-rated plant-based product per category; the *plant-based average*, defined as the average per category; and the *animal benchmark*, using Wilcoxon signed-rank tests. Statistical significance was reported based on two-sided *p*-values. We grouped the results of the overall liking ratings into *promoters*, including like very much (7) and like (6), *passive*, including like somewhat (5) and neither like nor dislike (4), and *detractors*, including dislike somewhat (3), dislike (2), and dislike very much (1). Participants also completed check-all-that-apply (CATA) questions that covered flavor, texture, and appearance attributes, from which we calculated attribute prevalence and conducted penalty analysis with mean drop and lift to quantify associations between attributes and overall liking. To relate sensory performance to commercial adoption, we analyzed 2024 US retail sales data from publicly available industry reports, retail scanner summaries, and NECTAR category analyses [25]. We defined plant-based market share as plant-based category sales divided by total category sales, including both plant-based and animal-based products, and defined sensory performance as the percentage of consumers who rated the plant-based product as the same or better than the animal benchmark in paired overall liking comparisons. All comparisons reflect paired within-subject sensory assessments because participants evaluated products within the *same* category under blinded conditions. The study aimed to benchmark category-level sensory performance rather than precisely rank individual formulations; accordingly, we aggregated responses across products within each category to reduce sensitivity to product-specific variability. The statistical analyses focused on predefined category-level benchmark comparisons rather than testing a single global hypothesis across all categories. Because the objective was comparative benchmarking, not confirmatory inference, we did not apply a formal multiple-comparison correction. Accordingly, *p*-values should be interpreted in conjunction with effect magnitude, consistency across categories, and practical sensory relevance rather than as standalone measures of significance.

## 3. Results

**Figure 3:**
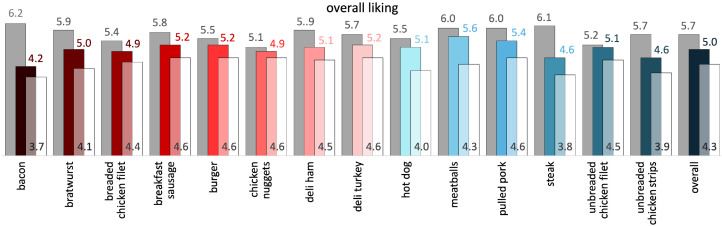
**Overall liking scores.** Mean overall liking scores of animal benchmark (gray), plant-based leader (dark color), and plant-based average (white) across all 14 product categories. Approximately 100 participants evaluated each product (mean per test = 99.4) on a seven-point Likert scale from dislike very much (1) to like very much (7). The overall mean across all 14 categories was 5.7 for animal benchmark, 5.0 for plant-based leader, and 4.3 for plant-based average (right).

In all 14 product categories, the animal benchmark received the highest overall liking scores, the plant-based leader received the second highest overall liking scores, and the plant-based average ranked lowest (Figure 3). The taste gap between the animal benchmark and the leading plant-based product was smallest in unbreaded chicken filets (Δ=0.1) and chicken nuggets (Δ=0.2). The taste gap was largest for bacon (Δ=2.0) and steak (Δ=1.5). The mean overall liking was 5.7 for the animal benchmark, 5.0 for the plant-based leader, and 4.3 for the plant-based average, which shows a clear gradient in consumer acceptance. These results indicate that leading plant-based products can approach animal benchmarks in overall liking in selected categories, but they do not yet match animal products on overall liking at the category level. The effect size analysis across approximately 100 paired comparisons per category (n=98.6±8.6, total n=1381) revealed negligible to small effects for unbreaded chicken filets (r=0.05), chicken nuggets (r=0.07), and burgers (r=0.19) but large effects for steak (r=0.62) and bacon (r=0.76). These effect sizes support the conclusion that some categories are approaching sensory parity while others are not (Table 1).

**Figure 4:**
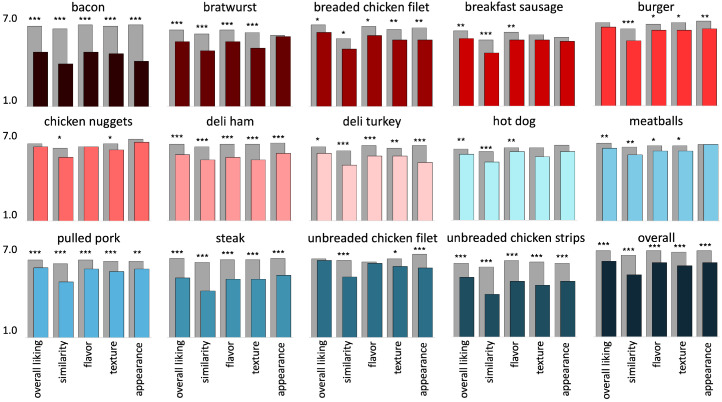
**Overall liking, similarity, flavor, texture, and appearance scores.** Performance of animal benchmark (gray) and plant-based leader (dark color) across all 14 product categories: bacon, bratwurst, breaded chicken filets, breakfast sausages, burgers, chicken nuggets, deli ham, deli turkey, hot dogs, meatballs, pulled pork, steak, unbreaded chicken filets, and unbreaded chicken strips. Scored by mean overall liking, similarity, flavor, texture, and appearance. Approximately 100 participants evaluated each product (mean per test = 99.4) on a seven-point Likert scale from dislike very much (1) to like very much (7). Across all 5 × 14 rankings, in 11 comparisons, there was no significant difference between animal benchmark and plant leader; in 59 comparisons, the animal benchmark scored significantly higher than the plant-based leader; ^∗∗∗^ *p* < 0.001, ^∗∗^ *p* < 0.01, ^∗^ *p* < 0.05.

Across 70 category-by-attribute comparisons, the animal benchmarks scored significantly higher than the plant-based leaders in 59 cases, while 11 cases showed no significant comparisons (Figure 4). No comparisons favored the plant-based products. This pattern was observed across all the attributes, suggesting that the performance gap remains broad rather than limited to single attributes. Similarity showed some of the largest and most consistent gaps across the categories, which suggests that the plant-based products struggle to closely replicate the animal benchmarks. The eleven cases that showed no significant differences were spread across four attributes (overall liking, flavor, texture, and appearance) and seven categories (bratwurst, breakfast sausages, burgers, pulled pork, hot dogs, meatballs and unbreaded chicken filets). For attributes that showed significant differences, the effect magnitudes also varied by category and attribute, from unbreaded chicken filets Δ=0.1 to bacon Δ=2.0, suggesting that certain animal products may be more successfully replicated than others. These results show that leading plant-based products can approach animal benchmarks in specific category–attribute comparisons, but, overall, the animal benchmark maintains an advantage across all the categories.

**Figure 5:**
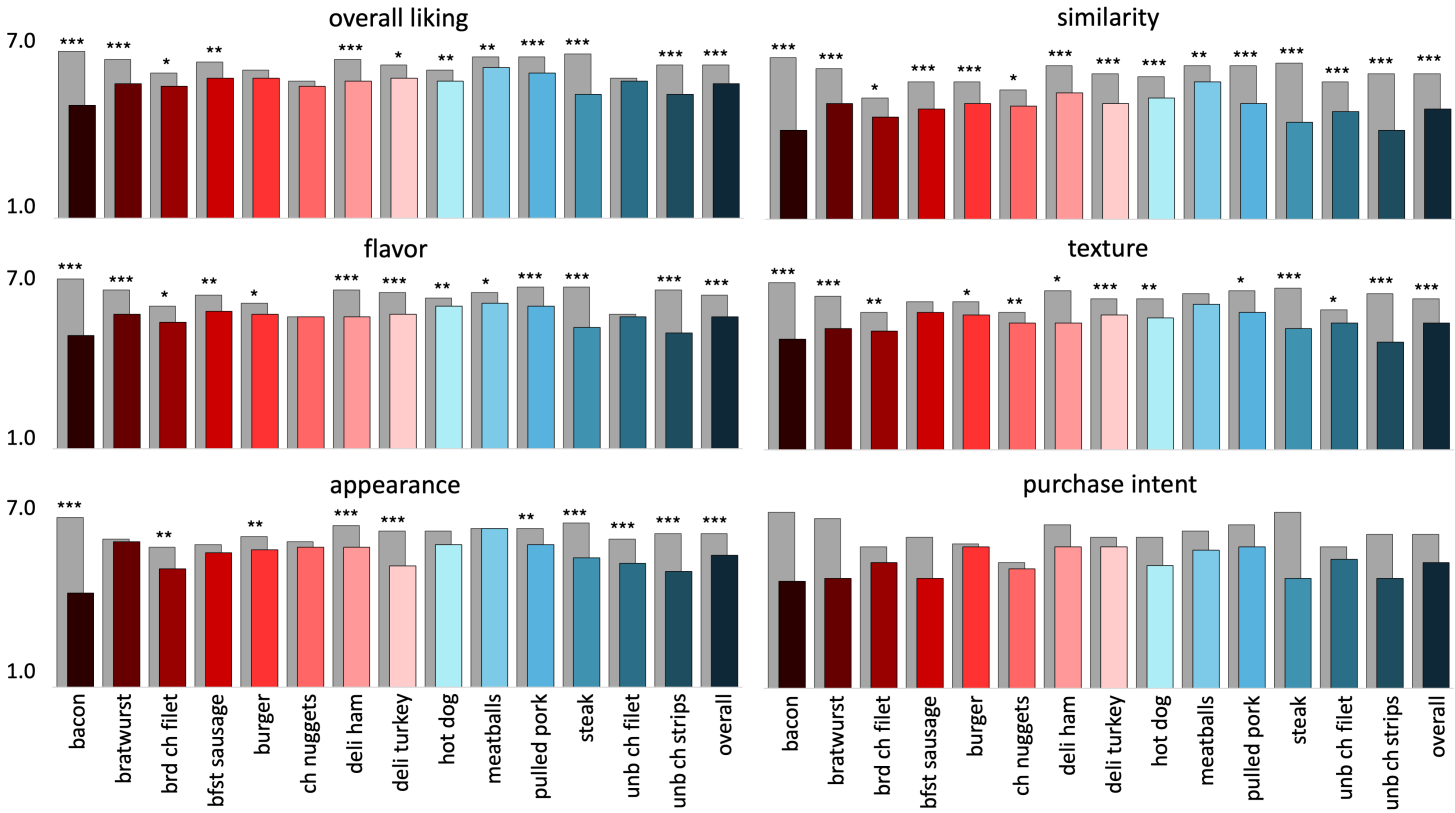
**Overall liking, similarity, flavor, texture, appearance, and purchase intent scores.** Performance of animal benchmark (gray) and plant-based leader (dark color) scored by mean overall liking, similarity, flavor, texture, appearance, and purchase intent across all 14 product categories: bacon, bratwurst, breaded chicken filets, breakfast sausages, burgers, chicken nuggets, deli ham, deli turkey, hot dogs, meatballs, pulled pork, steak, unbreaded chicken filets, and unbreaded chicken strips. Approximately 100 participants evaluated each product (mean per test = 99.4) on a seven-point Likert scale from dislike very much (1) to like very much (7). Across all 5 × 14 rankings, in 11 comparisons, there was no significant difference between animal benchmark and plant leader; in 59 comparisons, the animal benchmark scored significantly higher than the plant-based leader; ^∗∗∗^ *p* < 0.001, ^∗∗^ *p* < 0.01, ^∗^ *p* < 0.05.

Consistent with the sensory and liking difference (Figure 4), purchase intent for the animal benchmark was numerically greater than the leading plant-based product across all 14 categories (Figure 5). Thus, the performance gap was not limited to product evaluation but extended to stated consumer willingness to purchase. The animal-to-plant-based difference varied across categories, including bacon (Δ=2.2) and steak (Δ=2.1), with the largest difference for chicken nuggets (Δ=0.2) and burgers (Δ=0.1) having the smallest difference. This suggests that some product categories offer stronger commercial potential than others, although none reached parity with the animal benchmark.

**Figure 6:**
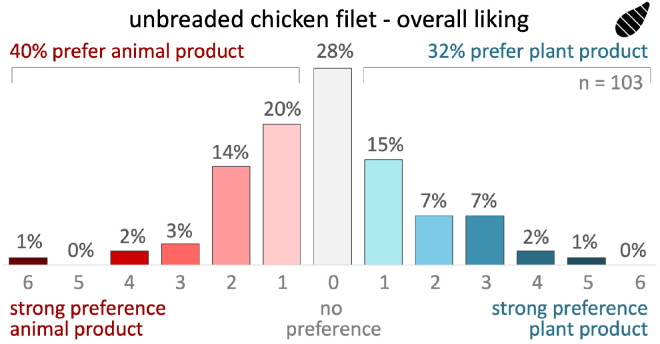
**Distribution of paired differences in overall liking between plant-based leader and animal benchmark.** Histogram of within-subject differences in seven-point overall liking score for Impossible Unbreaded Chicken Filets versus Perdue animal benchmark (n=103). Positive values indicate higher ratings for the plant-based product; negative values indicate higher ratings for the animal product; zero indicates no difference; 40% of participants preferred the animal benchmark, 32% preferred the plant-based product, and 28% reported no difference. The Wilcoxon signed-rank test did not detect a statistically significant directional preference between the plant-based and animal products, *p* = 0.314.

The best-performing plant-based category was unbreaded chicken with the Impossible Unbreaded Chicken Filets as the plant-based leader (Figure 6). We calculated the within-subject differences in overall liking by subtracting each participant’s rating of the animal product from their rating of the plant-based product (n=103). Indeed, 40% of the respondents rated the animal benchmark higher, 32% rated the plant-based product higher, and 28% reported no preference. The histogram also shows that many participants clustered near small differences, which indicates that many respondents considered the products as close in overall liking. Although the animal products received slightly higher overall liking ratings, the within-subject comparisons were broadly split, with over one-quarter of the respondents indicating no preference. The Wilcoxon signed-rank test did not detect a statistically significant directional preference between the plant-based and animal products (*p* = 0.314). These results suggest that, for this product category, the leading plant-based product approached the animal benchmark sufficiently closely to support the technical feasibility of taste parity.

**Figure 7:**
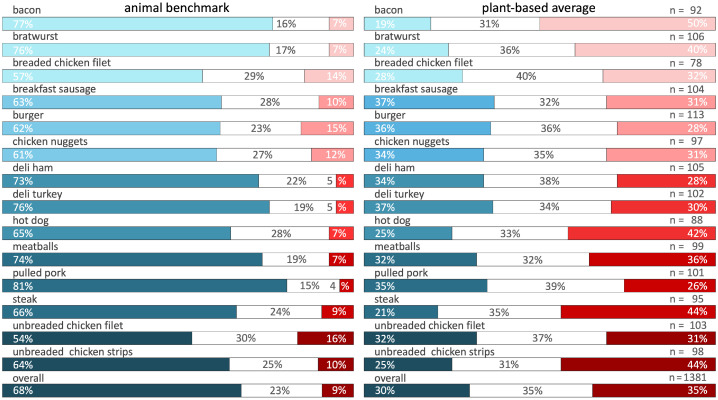
**Promoter, passive, and detractor shares for plant-based averages and animal benchmarks across categories.** Stacked bars show the percentage of responses classified as promoters (like very much and like), passives (like somewhat and neither like nor dislike), and detractors (dislike somewhat, dislike, and dislike very much) for each product category (n=98.6±8.6 per category). Across all categories, plant-based averages (right) show lower promoter shares and higher detractor shares than animal benchmarks (left). Overall, 30% of responses were classified as promoters for the plant-based average compared with 68% for animal benchmarks, while detractor shares reached 35% for plant-based products compared with 9% for animal products.

The overall liking ratings show clear differences between the plant-based averages and animal benchmarks across all the categories (Figure 7). We classified responses as promoters (like very much and like), passives (like somewhat and neither like nor dislike), and detractors (dislike somewhat, dislike, and dislike very much) based on seven-point overall liking ratings. Across all the categories, plant-based products generated substantially fewer promoters and more detractors than animal benchmarks. On average, 30% of the respondents rated the plant-based product as like very much or like, whereas 68% rated the animal benchmark in these top two categories. In contrast, 35% rated plant-based products in one of the dislike categories compared with 9% for animal products. The data also reveal wide variation across plant-based categories. Categories such as pulled pork, deli turkey, burgers, and breakfast sausages achieved promoter shares in the mid-30% range, whereas bacon and steak filets showed promoter shares near or below 20% and detractor shares exceeding 40%. Taken together, these patterns highlight both the magnitude of the liking gap relative to the animal benchmark and the uneven performance of plant-based products across categories.

**Figure 8:**
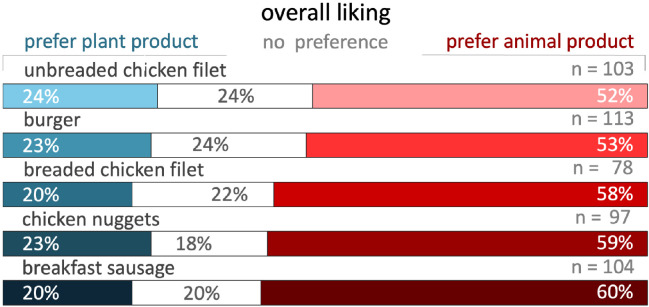
**Preference comparison between plant-based leader and animal benchmark in top-performing categories.** Stacked bars show the percentage of participants who preferred the plant-based leader, reported no preference, or preferred the animal benchmark based on within-subject overall liking comparisons (n=99.0±13.4 per category). Across the five highest-performing categories, at least 40% of participants rated the plant-based product the same as or better than the animal benchmark. Unbreaded chicken filets (48%) and burgers (47%) achieved the highest shares of same or better ratings, followed by breaded chicken filets (42%), nuggets (41%), and breakfast sausages (40%).

Five plant-based categories performed well against the animal benchmark (Figure 8). For each category, we inferred relative preference from paired within-subject overall liking comparisons for the plant-based product against its animal benchmark. Across these five categories, at least 40% of the participants rated the plant-based product the same as or better than the animal benchmark. Unbreaded chicken filets (48%) and burgers (47%) achieved the highest shares of same or better evaluations, indicating near-parity for nearly half of the participants. Breaded chicken filets (42%), nuggets (41%), and breakfast sausages (40%) also demonstrated substantial competitive performance. Although a majority of the participants still preferred the animal benchmark in each category, these results show that a meaningful segment of consumers do not perceive a clear disadvantage in several plant-based formats.

**Figure 9:**
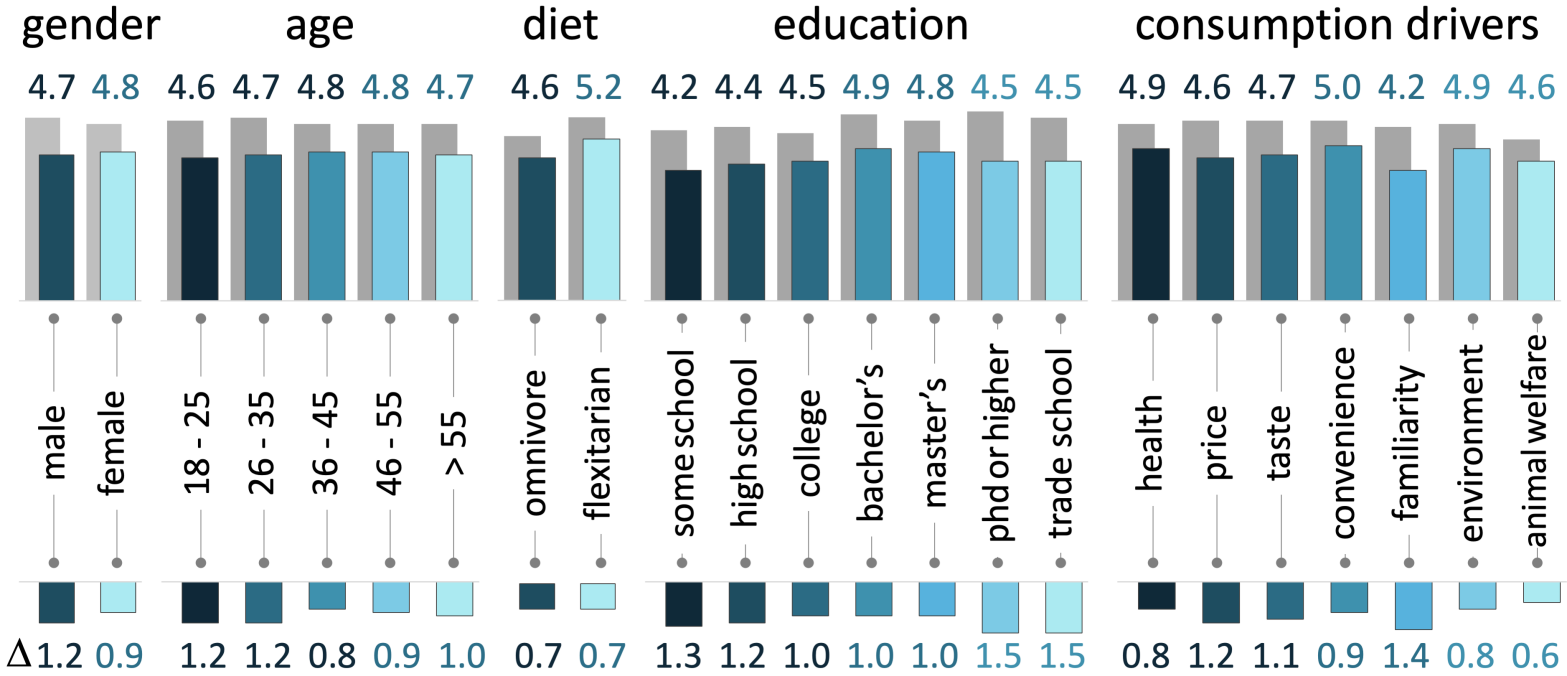
**Mean purchase intent across demographic segments and consumption drivers.** Mean purchase intent on a seven-point Likert scale from definitely would not buy (1) to definitely would buy (7) across gender, age, dietary preference, education, and self-reported consumption drivers for animal benchmark (gray) and plant-based (blue) products (top bars) and difference Δ between them (bottom bars).

The mean purchase intent for animal benchmark products exceeds that of plant-based products across all the demographic segments and consumption drivers (Figure 9). The differences between animal benchmark and plant-based products ranged from 0.5 to 1.5 points on the seven-point scale. The smallest gap appears in the consumption driver of animal welfare (5.2 vs. 4.6; Δ = 0.5), whereas larger gaps appear among respondents with PhD or trade school education (6.0 vs. 4.5 and 6.0 vs. 4.5; Δ = 1.5). Females and middle-aged consumers (36–55) showed relatively higher purchase intent for plant-based products compared with other demographic groups. Among consumption drivers, respondents prioritizing health and environment exhibited smaller differences (Δ = 0.8), whereas those prioritizing taste, price, and familiarity showed larger preference gaps favoring animal meat (Δ = 1.1 to 1.4). Taken together, these results indicate stronger receptivity among health- and environment-oriented consumers and middle-aged segments and comparatively lower receptivity among younger consumers and those who emphasize taste, price, and familiarity.

**Figure 10:**
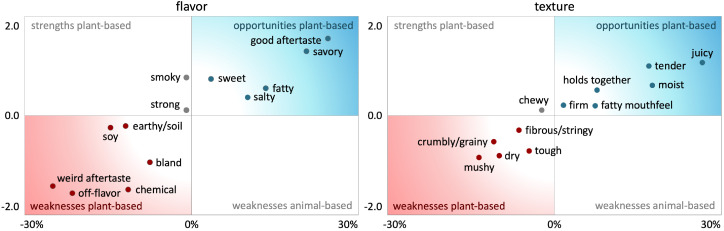
**Penalty analysis of flavor and texture attributes and R&D opportunities for plant-based products.** Net prevalence as percentage selecting attribute for animal minus plant-based average plotted against impact on overall liking as mean lift/penalty on a seven-point Likert scale based on check-all-that-apply responses. Attributes in the upper-right quadrant indicate opportunities for plant-based products; attributes in the lower-left quadrant indicate plant-based weaknesses. For flavor, the largest opportunities include improving aftertaste and savoriness and reducing off-flavors and chemical notes (left). For texture, the strongest opportunities include increasing juiciness and tenderness and reducing mushiness, dryness, and crumbliness (right).

The penalty analysis identified clear sensory priorities for plant-based product improvement (Figure 10). We quantified each attribute by combining its relative prevalence (animal minus plant-based average) with its impact on overall liking. The attributes in the upper-right quadrant represent high-value opportunities because they occur more frequently in animal products and increase liking. In flavor, improving aftertaste and increasing savoriness produced the largest positive effects, while reducing off-flavors and chemical notes also yielded substantial gains. Moderate opportunities included increasing fattiness and saltiness and reducing blandness (left). In texture, increasing juiciness showed the strongest positive association with liking, followed by tenderness and moistness. Reducing mushiness, dryness, and crumbliness also improved liking and addressed attributes that consumers more often associated with plant-based products (right). Taken together, these results define a prioritized sensory roadmap that links specific attribute gaps to measurable gains in overall liking.

**Figure 11:**
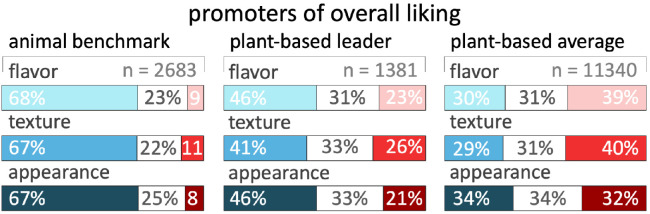
**Promoter, passive, and detractor shares for flavor, texture, and appearance across product tiers.** Stacked bars show the percentage of responses classified as promoters (like very much and like), passives (like somewhat and neither like nor dislike), and detractors (dislike somewhat, dislike, and dislike very much) for each product category for the animal benchmark (n=2683), plant-based leader (n=1381), and plant-based average (n= 11,340).

The promoter, passive, and detractor shares for flavor, texture, and appearance across the animal benchmark, plant-based leader, and plant-based average suggest that improvements to flavor and texture should be prioritized (Figure 11). We classified responses as promoters (like very much and like), passives (like somewhat and neither like nor dislike), and detractors (dislike somewhat, dislike, and dislike very much) based on seven-point overall liking ratings. For flavor, the share of promoters increases from 30% for the plant-based average to 46% for the plant-based leader, approximately a 1.5× fold increase, and represents the largest promoter gain across the three attributes. For texture, we observe the largest opportunity for the plant-based leader with 41% promoters to close the remaining gap to the animal benchmark with 67% promoters. For appearance, we identify the lowest R&D priority because the plant-based average receives better promoter scores for appearance with 34% than for flavor with 30% and texture with 29%.

**Figure 12:**
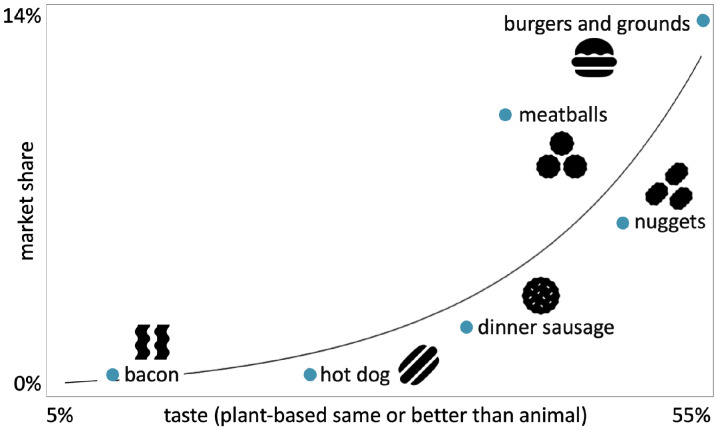
**Relationship between market share and sensory performance across plant-based categories.** Scatterplot shows plant-based market share (percentage of plant-based products of 2024 total retail sales, including both plant-based and animal-based) as a function of sensory performance (percentage of consumers rating plant-based products same or better than animal benchmark in overall liking). Categories with higher sensory parity exhibit substantially greater market penetration. Burgers and grounds, meatballs, and nuggets achieve both higher taste parity (≈40–55%) and higher market share (≈6–14%), whereas lower-performing categories in taste parity, such as bacon and hot dogs (≈10–25%), only capture a small fraction of category sales (≈1%).

Our results reveal a strong positive association between sensory performance and plant-based market share across categories (Figure 12). We defined sensory performance as the percentage of consumers who rated plant-based products as the same or better than the animal benchmark in overall liking and defined market share as plant-based sales divided by total category sales, plant-based and animal-based, in 2024 retail data [25]. Categories with a higher taste parity captured substantially greater market penetration. Burgers and grounds achieved the highest taste parity and the highest market share (≈55% parity; ≈14% share), followed by meatballs (≈40% parity; ≈10% share) and nuggets (≈50% parity; ≈6% share). In contrast, bacon and hot dogs achieved substantially lower taste parity and only a marginal faction of sales (≈10–25% parity; less than 1% share). The fitted curve illustrates a positive association between improved sensory performance and category-level penetration. Taken together, these results indicate that categories that approach sensory parity secure a disproportionately greater share from the animal segment, whereas categories that fail to match animal taste remain commercially constrained.

## 4. Discussion

This study presents one of the largest blinded in-person sensory evaluations of plant-based meat products to date, with 2684 consumers evaluating products across 14 categories, resulting in more than 11,000 product evaluations and more than 800,000 data points. Compared to our previous study with 1150 consumers evaluating products across five categories in a test kitchen setting [24], the current study more than doubled the number of participants, more than doubled the number of categories, and used authentic restaurant locations [25]. At the category average level, plant-based products continue to lag behind animal benchmarks in overall liking, which aligns with prior work documenting persistent sensory gaps [14,26,27]. At the same time, our results show that taste parity lies within reach. Several plant-based products achieve strong overall liking, and some show no statistically significant difference from animal benchmarks in direct comparison. These findings support emerging evidence that next-generation plant-based meats can narrow historical acceptance gaps when manufacturers prioritize sensory performance [20,21].

**Leading products highlight strong potential to meet taste expectations.** Our data show that taste parity no longer represents an abstract goal. In four cases, the animal benchmark does not achieve a statistically significant preference over a leading plant-based product. At the category level, five plant-based categories—unbreaded chicken filets, burgers, breaded chicken filets, nuggets, and breakfast sausages—achieve the same or better ratings from at least 40% of the participants. Unbreaded chicken filets and burgers approach parity for nearly half of the respondents. Prior research demonstrates that consumers judge plant-based meats in direct comparison to familiar animal analogs, [14,26], and our findings indicate that several categories now compete meaningfully on taste within that comparative frame. Plant-based burgers continue to attract substantial scientific and commercial attention [28,29], and our results show that their overall liking is almost on par with that of the animal counterpart. Because we defined the plant-based leader as the highest-rated product within each category, these comparisons intentionally represent best-case category performance and therefore likely overestimate the average sensory competitiveness of commercially available plant-based products. The substantially lower scores of the plant-based category averages highlight that sensory parity remains uneven across the broader market despite the emergence of strong individual products.

**Improvement is needed, worthwhile, and attainable.** Despite the strength of the category leaders, the typical plant-based product continues to underperform. Across categories, the plant-based averages generate more detractors than promoters, while the animal benchmarks show the opposite pattern. Earlier research shows that taste satisfaction drives repeat purchase and long-term substitution [14,18,30], even in hypothetical choice experiments [31], and our results reinforce that conclusion. We identify clear whitespace in categories such as bacon, steak, and unbreaded chicken strips, where detractor shares remain high. At the same time, categories that achieve higher taste parity capture substantially greater market penetration. Burgers, nuggets, and meatballs reach market shares between 5% and 14%, whereas lower-performing categories such as bacon and hot dogs remain below 1%. Leading products also achieve nearly double the promoter share of category averages. These results show that substantial sensory improvement remains technically achievable, although broader adoption will also depend on factors such as familiarity, availability, and socio-cultural norms [32]. Future work could further resolve heterogeneous consumer subgroups through clustering or latent preference modeling to identify category-specific acceptance patterns.

**Closing the sensory gap requires decoding flavor and structure.** Flavor emerges as the dominant perceptual differentiator, while texture remains the most persistent structural gap between plant-based and animal products [32]. Participants describe plant-based products as less savory and report off-flavors or atypical aftertastes more frequently than for animal benchmarks. Prior sensory research identifies flavor authenticity and textural realism as central determinants of meat acceptance [20,26], and our data reinforce this conclusion across multiple categories. Texture deficits, particularly reduced juiciness and altered tenderness, further diminish liking, consistent with long-standing evidence that mouthfeel and mechanical response shape perceived quality [16,19]. These findings underscore a fundamental scientific challenge: quantitatively connecting formulation, microstructure, rheology, and multisensory perception [23,33]. Meat perception does not arise from a single attribute but from the integration of chemical composition, mechanical response, fat–water distribution, and flavor–aroma interactions [34]. Bridging this gap requires models that relate measurable physical properties to sensory outcomes and capture nonlinear multivariate dependencies across ingredients, processing conditions, and human perception [35]. Future work could further resolve these interactions through structural equation modeling and causal sensory frameworks.

**Artificial intelligence enables quantitative integration of structure and perception.** Recent advances in artificial intelligence offer a framework to address this complexity [36]. Multimodal learning can integrate ingredient lists, nutritional vectors, rheological measurements, and sensory descriptors into shared latent representations that enable both prediction and inverse design [17]. When paired with large-scale blinded sensory evaluations, such approaches move beyond trial-and-error reformulation toward systematic data-driven understanding and generative artificial intelligence for systematic formulation design [37]. Our results therefore extend beyond benchmarking current products. By curating and openly sharing more than 11,000 comparative sensory evaluations across 14 categories, we provide structured labeled data that can support quantitative modeling of sensory performance at scale [25]. Adoption of sustainable proteins will depend in part on achieving robust sensory equivalence, as prior behavioral studies consistently demonstrate [18]. However, increased awareness alone does not ensure dietary change [38], and long-term adoption will likely also depend on economic, cultural, and behavioral factors beyond sensory performance. Achieving this goal requires mechanistic insight rather than incremental iteration. Integrating sensory science, quantitative mechanics, and artificial intelligence creates a pathway to decode what makes meat meaty and to translate that knowledge into principled advances in sustainable protein design.

## 5. Conclusions

This study delivers one of the largest blinded in-person sensory comparisons of plant-based and animal meat products to date and clarifies where the field stands today. First, most plant-based products still require significant research and development. Across 14 categories, the plant-based average achieved 30% promoters compared with 68% for the animal benchmarks and generated nearly four times as many detractors. Second, leader products have emerged in most categories. In unbreaded chicken filets, burgers, nuggets, breaded chicken filets, and breakfast sausages, at least 40% of the participants rated the plant-based leader the same as or better than the animal benchmark. Third, taste parity is achievable. In unbreaded chicken filets, the within-subject comparison revealed no statistically significant preference for the animal product (*p* = 0.314), and the overall liking gap reached only 0.1–0.2 points in the best-performing categories. Fourth, sensory performance strongly correlates with commercial performance: categories that approach taste parity, such as burgers, nuggets, and meatballs, secure a 5–14% market share, whereas low-parity categories such as bacon and hot dogs remain below 1%. Finally, the path to parity requires targeted improvements in flavor and texture. Penalty analysis identifies savoriness, aftertaste, juiciness, and tenderness as the most powerful levers to increase liking, while appearance plays a secondary role relative to flavor and texture.

Taken together, these findings show that plant-based meat has moved beyond proof of concept. The category now contains clear sensory leaders and commercially viable formats, yet it has not achieved consistent mainstream equivalence across categories. Plant-based meat stands at a transitional moment: technical feasibility exists, category-level performance varies widely, and systematic sensory optimization can close the remaining gap. To accelerate progress, we have made all the sensory, preference, and market-linked data from this study publicly available. By democratizing access to large-scale blinded sensory evidence, we aim to enable independent validation, stimulate rigorous food science, and accelerate discovery and innovation across the sustainable protein ecosystem.

## Figures and Tables

**Table 1:** **Overall liking scores.** Comparisons between animal benchmark and plant-based leader across all 14 product categories. Values are reported as mean ± standard deviation. Difference Δ denotes the paired difference in overall liking score. Statistical significance is assessed using Wilcoxon signed-rank tests. Effect sizes are calculated as $r=Z/\sqrt{n}$, where Z is the standardized Wilcoxon signed-rank test statistic and *n* is the number of paired observations.

Category	Respondents n	Plant-Based Leader	Animal Benchmark	Difference Δ	Significance p	Effect Size r
bacon	92	4.15±1.78	6.23±1.00	2.08±1.77	<0.001	0.76
bratwurst	106	5.03±1.51	5.90±1.35	0.87±1.90	<0.001	0.43
breaded chicken filets	78	4.87±1.59	5.38±1.48	0.51±1.99	0.024	0.25
breakfast sausages	104	5.16±1.63	5.80±1.32	0.63±1.94	<0.001	0.33
burgers	113	5.15±1.60	5.46±1.64	0.31±1.96	0.049	0.19
chicken nuggets	97	4.95±1.70	5.09±1.50	0.14±2.02	0.510	0.07
deli ham	105	5.07±1.60	5.88±1.40	0.81±1.91	<0.001	0.40
deli turkey	102	5.24±1.49	5.70±1.37	0.46±1.98	0.029	0.22
hot dogs	88	5.13±1.51	5.52±1.36	0.40±1.92	0.044	0.21
meatballs	99	5.58±1.44	6.03±1.09	0.45±1.58	0.005	0.28
pulled pork	101	5.44±1.37	6.04±1.34	0.60±1.82	<0.001	0.39
steak	95	4.58±1.80	6.07±1.27	1.49±2.06	<0.001	0.62
unbreaded chicken filets	103	5.12±1.40	5.19±1.58	0.08±1.81	0.625	0.05
unbreaded chicken strips	98	4.55±1.69	5.69±1.50	1.14±2.21	<0.001	0.47

## Data Availability

All data are freely available at https://www.nectar.org/sensory-research/2025-taste-of-the-industry (accessed on 10 May 2026).
